# Effects of Prehabilitation by Physical Activity on Fatigue After Surgery for Colorectal Cancer. Results From Secondary Analyses in the Randomized Controlled Trial PHYSSURG‐C

**DOI:** 10.1002/cam4.72142

**Published:** 2026-08-08

**Authors:** Kevin Afshari, Aron Onerup, Ying Li, Eva Angenete, Carolina Ehrencrona, Anette Wedin, Eva Haglind

**Affiliations:** ^1^ Scandinavian Surgical Outcomes Research Group (SSORG), Department of Surgery, Institute of Clinical Sciences, Sahlgrenska Academy University of Gothenburg Gothenburg Sweden; ^2^ Department of Surgery Sahlgrenska University Hospital Gothenburg Region Vastra Götaland Sweden; ^3^ Department of Pediatrics, Institute of Clinical Sciences, Sahlgrenska Academy University of Gothenburg Gothenburg Sweden; ^4^ Department of Pediatric Oncology Sahlgrenska University Hospital Gothenburg Region Vastra Götaland Sweden

**Keywords:** colorectal cancer, fatigue, prehabilitation, surgery

## Abstract

**Objective:**

The objective of this study is to explore effects on self‐reported fatigue at 4 weeks and 12 months after surgery in patients with colorectal cancer undergoing a pre‐ and postoperative exercise intervention compared to standard care in a randomized trial.

**Background:**

Fatigue after cancer surgery is common and evidence suggests it could be alleviated by physical activity. In this post hoc exploratory study, we analyzed fatigue and other secondary outcomes from a randomized trial aimed at exploring if prehabilitation by additional physical activity could improve postoperative recovery.

**Method:**

PHYSSURG C was a multicenter, controlled, randomized trial. Patients diagnosed with colorectal cancer were randomized to additional unsupervised moderate physical exercise in accordance with WHO guidelines or usual care two weeks before and four weeks after surgery. Trial‐specific questionnaires were used at baseline, at 4 weeks and 12 months. Outcomes were self‐reported fatigue as well as quality of life, pain, and mental recovery at both postoperative follow‐up times.

**Results:**

A total of 518 patients were included in this analysis. At 4 weeks after surgery, the mean difference in fatigue was 3.75 ((CI 0.01;7.49), *p* = 0.05) with intention to treat approach, and in a per protocol analysis at 4 weeks the mean difference was 4.04 ((CI 0.14;7.93), *p* = 0.04), and at 12 months it was 4.29 ((CI 0.44; 8.14), *p* = 0.03). There were no differences between the intervention and control groups regarding quality of life, pain, or mental recovery.

**Conclusion:**

This exploratory study found a suggestion of less postoperative fatigue in the intervention group. Further studies are needed to define the role of unsupervised moderate exercise, if any, before and after surgery in regard to postoperative fatigue in patients with colorectal cancer.

**Trial Registration:**

ClinicalTrials.gov identifier: NCT02299596

## 1 Introduction

The World Health Organization has reported that low levels of physical activity influence the risk of developing cancer, including colorectal cancer. For general health purposes, including the risk of malignant tumors, WHO recommends 150–300 min per week of aerobic physical activity at a medium level for adults.

Prehabilitation before surgery has been tested for different types of abdominal cancer, but a systematic review found that considerable differences in prehabilitation programs of physical activity with or without other components made it impossible to reach a firm overreaching conclusion [1]. For patients diagnosed with colorectal cancer, prehabilitation before treatment has been hypothesized to improve outcomes, such as physical strength, recovery and risk of postoperative complications. As described in a recent meta‐analysis on unimodal high or moderate intensity training in patients with colorectal cancer studies have come to different results [2]. Randomized trials with multicomponent interventions in frail patients with major abdominal or colorectal cancer surgery have reported conflicting results on impact on complications after surgery [3, 4]. In PHYSSURG‐C, a randomized, controlled, multicenter superiority trial with the aim to improve short term recovery, an intervention of increased physical activity before surgery for colorectal cancer as well as after discharge, found no difference in self‐reported physical recovery at four weeks postoperatively in the intervention group compared to routine care [5].

Cancer treatment often leads to fatigue, a state of tiredness that is not improved by resting. A review of 11 studies with low level of bias reported improvement of fatigue after supervised exercise training [6]. In a multicentre international cohort of patients operated for rectal cancer, we found an association between pain and fatigue with global quality of life [7]. Reviews suggest that fatigue and mental health can improve after physical training, specifically in patients with cancer [8, 9]. In patients treated with adjuvant chemotherapy after surgery for breast cancer, a program of high‐intensity interval and resistance training was found superior to usual care regarding fatigue, pain, and emotional function [10].

The aim of this exploratory study was to analyze secondary endpoints in the randomized trial PHYSSURG‐C. The primary aim was to investigate the extent to which the effects on self‐reported fatigue at 4 weeks and 12 months after surgery differed between colorectal cancer patients who underwent a pre‐ and postoperative exercise intervention compared to standard care. Secondary objectives were to investigate the effects of the intervention on self‐reported quality of life, pain, and mental recovery.

## 2 Methods

The PHYSSURG‐C trial was a randomized trial comparing the effect of an intervention of pre‐ and postoperative physical activity with standard care on postoperative recovery after colorectal cancer surgery. The methods have been described previously [11]. In summary, the design was a prospective, randomized, controlled, open‐label, superiority multicenter trial set in Sweden. The intervention was an individualized program of physical activity including aerobic exercise of relative medium intensity 30 min per day according to the WHO recommendation for adults and inspiratory muscle training 30 × 2, twice daily. The intervention was intended to be performed 14 days before surgery, followed by four weeks with only aerobic exercise after discharge from hospital.

Six Swedish hospitals included patients from January 22, 2015 until May 28, 2020. All patients fulfilling the inclusion criteria (20 years or older, elective surgery for colon or rectal cancer, understanding of Swedish) were eligible for participation. Exclusion criteria were emergency surgery, local excision, cytoreductive surgery with Hyperthermic Intraperitoneal Chemotherapy (HIPEC) or inability to perform the intervention (physical inability or short time until surgery).

Patients were approached after diagnosis and in adjunction to an outpatient hospital visit at a participating surgical department. After informed consent, randomization was performed using a digital system that created the sequence, unknown to the research nurse, with a block size of four. Stratification was by type of surgery (laparoscopic/open), tumor location (colon/rectum), and neoadjuvant therapy (rectum with radio(chemo)therapy prior to surgery/rectum without radio(chemo)therapy prior to surgery).

### 2.1 Intervention

Each participant in the intervention group met with a physiotherapist at the time of inclusion. to discuss individually suitable physical activity and to receive instructions about inspiratory muscle training, including how to adjust the resistance in the device used for this exercise. The physiotherapist would listen to descriptions from the patient of types of physical activity they normally would undertake, and depending on this, would typically recommend walking, running, swimming, or bicycling for aerobic exercise, of an intensity to result in breathlessness but leaving the individual able to talk.

One week into the preoperative period all patients in the intervention group were contacted by phone, reminded about changing the resistance in the device for inspiratory muscle training and encouraged to adhere to the aerobic training and again three weeks into the postoperative period to encourage aerobic physical activity.

### 2.2 Data gathering

Baseline clinical data retrieval through a case report form (CRF) was made by research nurses at the time of inclusion. Clinical outcomes were retrieved from hospital records by two of the authors using a prespecified CRF. Details about the tumor, American Society of Anesthesiologists classification (ASA), radicality, surgical technique, and pathology reports were retrieved from the Swedish ColoRectal Cancer Register (SCRCR). The SCRCR has high validity regarding the variables used [12]. Questionnaires were analogue and returned to the secretariate by a pre‐paid envelope. The patients received the baseline questionnaire at inclusion to be filled out at home. At follow‐ups (4 weeks and 12 months after surgery), a research nurse contacted the patients by phone before sending the questionnaire. Patients in the intervention group registered physical activity during the two weeks period before surgery in a diary.

### 2.3 Outcomes

Fatigue was determined at baseline, 4 weeks and 12 months postoperatively through the vitality domain in the RAND‐36 instrument which was part of the questionnaires. All questions were set to cover the situation during the past 4 weeks. The vitality domain is one of eight subscales in RAND. It is based on 4 questions and was calculated according to the Swedish manual 1.02. This gives a vitality scale with a range of 0–100 where low scores indicate feeling worn out while high scores indicate feeling full of energy. The Swedish translation of RAND‐36 has been found content equivalent to the original instrument [13] and the internal consistency reliability was above 0.8 with Cronbach's alpha [14].

Quality of life at baseline, 4 weeks and 12 months postoperatively was measured using the vertical visual analogue scale (VAS) in the EQ‐5D‐3L. The scale ranges from 0 (worst imaginable health) to 100 (best imaginable health). The EQ‐5D is a frequently used generic health related quality of life measurement [15].

Pain at 4 weeks and 12 months postoperatively was measured by Brief Pain Inventory, Short Form (BPI‐SF). The BPI was developed as a pain assessment tool for cancer patients [16]. We used the reactive dimension, i.e., how the pain interfered with patients' daily life. The pain interference is based on seven questions. It was calculated as the arithmetic mean of the interference items when at least four of the seven items were completed, according to recommendations [17]. The BPI‐SF has demonstrated high internal consistency across cancer patient populations in several countries and translation with a Cronbach alpha reliability ranged 0.77–0.91 and for the pain interference items 0.89 to 0.92 [17, 18].

Mental recovery was measured at 4 weeks and 12 months postoperatively, using the following question “To what extent do you feel mentally recovered after the operation?”. This question was developed within the research group.

### 2.4 Statistics

Sample size for the primary outcome in PHYSSURG‐C “physical recovery” was calculated before the start of the inclusion based on a superiority hypothesis regarding the effect on recovery of the intervention studied. Due to the restricted knowledge of what to expect regarding effect size, a planned interim analysis with a recalculation of sample size was made by an external group after the first 100 randomizations, leading to an increase in sample size. One further increase in the sample size was made at a later stage when it was obvious that the drop‐out rate was larger than expected. Sample size was not calculated for secondary outcomes.

In these exploratory analyses of secondary outcomes, the analysis population consisted of all randomized participants who returned questionnaires at 12‐month follow‐up. The statistical analyses were performed according to a prespecified statistical analysis plan. The primary analyses were as randomized, that is by intention‐to‐treat principle. Per protocol analyses were performed for exploratory reasons to determine the effect of the intervention if performed. In these analyses, participants randomized to the intervention group who returned exercise diaries with reported preoperative exercise (inspiratory muscle training and/or aerobic exercise) during at least eight days were analyzed as having performed the intervention (per protocol) as described earlier [5, 19]. All participants randomized to control were analyzed as control. Participants randomized to intervention, but who did not return exercise diaries, or who returned diaries not meeting the criteria for compliance as described above were excluded from the per protocol analyses.

The descriptive information on outcomes of fatigue, quality of life, pain and self‐assessed mental recovery at follow‐up 4 weeks and 12 months are presented in tables. Linear mixed models were used to assess differences in fatigue, quality of life, and pain at 4 weeks and 12 months. Results are presented as mean differences, 95% confidence intervals (CI), and *p*‐values. Ordinal regression models were used for mental recovery at 4 weeks and 12 months separately. The binary treatment variable (control/intervention) was the primary variable of interest. Models were adjusted for baseline, time point (4 weeks/12 months), time‐treatment interaction, as well as stratification factors namely tumor site (colon/rectum), neoadjuvant therapy (none/radiotherapy/chemoradiotherapy), and surgery type (open/laparoscopic). Missing values were handled by a listwise deletion principle.

As these analyses of secondary outcomes were exploratory in nature, no correction of multiplicity was performed.

Statistical analyses were performed using R, version 4.3.2.

## 3 Results

**FIGURE 1:**
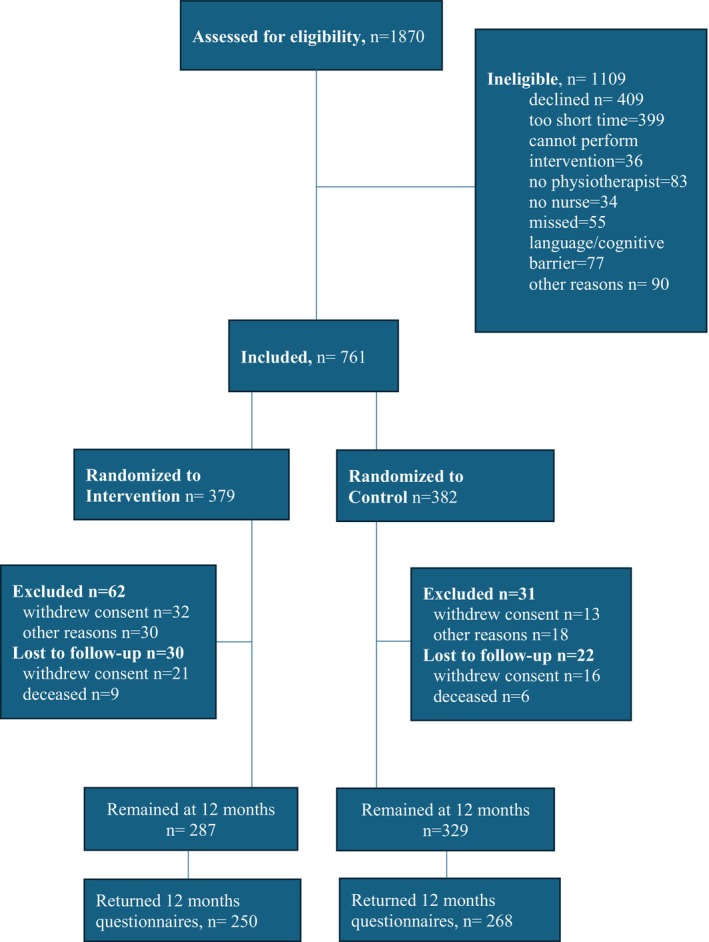
Flow chart.

A total of 518 patients completed and returned the questionnaires at follow up 12 months after surgery, 250 in the intervention group and 268 in the control group (Figure 1). The demography is described in Table 1.

**TABLE 1:** Demographic characteristics.

Characteristic	Intervention, *N* = 250	Control, *N* = 268
Age, median (Interquartile range)	70 (62, 75)	70 (62, 77)
Sex, *n* (%)		
Female	98 (39%)	111 (41%)
Male	152 (61%)	157 (59%)
BMI, median (Interquartile range)	25.4 (23.1, 28.4)	25.3 (22.8, 27.5)
Missing, *n*	0	1
Level of educatio*n*, *n*(%)		
Com*p*ulsory school	50 (21%)	55 (21%)
Upper secondary school	100 (41%)	119 (46%)
University	93 (38%)	87 (33%)
Missing, *n*	7	7
Ethnicity, *n*(%)		
Born abroad	33 (13%)	32 (12%)
Born in Sweden	212 (87%)	231 (88%)
Missing, *n*	5	5
Occupation, *n*(%)		
Retired	158 (66%)	168 (63%)
Sick leave/unemployed	13 (5.4%)	27 (10%)
Working	70 (29%)	70 (26%)
Missing, n	9	3
Marital status, *n*(%)		
Married or cohabiting	179 (73%)	199 (76%)
Single household	66 (27%)	64 (24%)
Missing, *n*	5	5
Smoking, *n*(%)		
Active regular smoker	6 (2.5%)	7 (2.7%)
No active regular smoking	237 (98%)	256 (97%)
Missing, *n*	7	5
Risk drinking, *n*(%)		
Risk drinking	32 (15%)	39 (18%)
No risk drinking	179 (85%)	177 (82%)
Missing, *n*	39	52
Level of physical activity at baseline, *n*(%)		
Sedentary	37 (15%)	44 (17%)
Some physical activity	164 (68%)	174 (67%)
Regular physical activity and training	39 (16%)	38 (15%)
Regular hard physical training	2 (0.8%)	4 (1.5%)
Missing, *n*	8	8
Comorbidity		
Cardiovascular disease	101 (40%)	101 (38%)
Diabetes mellitus	33 (13%)	30 (11%)
pulmonary disease	7 (2.8%)	9 (3.4%)
Neurological disease	6 (2.4%)	4 (1.5%)
Mental disease	16 (6.4%)	23 (8.6%)
Other disease	93 (37%)	109 (41%)
Tumor stage, *n*(%)		
0	0 (0%)	1 (0.4%)
I	70 (30%)	74 (30%)
II	58 (25%)	77 (31%)
III	89 (39%)	78 (31%)
IV	13 (5.7%)	19 (7.6%)
Missing, *n*	20	19
ASA classification, *n*(%)		
1	45 (20%)	45 (18%)
2	144 (63%)	154 (62%)
3	39 (17%)	49 (20%)
4	2 (0.9%)	0 (0%)
Missing, *n*	20	20
Type of surgery, *n*(%)		
Colon resection	116 (52%)	109 (45%)
Anterior resection	63 (28%)	80 (33%)
Abdominoperineal excision	46 (20%)	51 (21%)
Missing, *n*	25	28

Fatigue 4 weeks after surgery showed a mean difference between intervention and control groups of 3.75 (CI 0.01; 7.49), *p* = 0.05 and at 12 months the mean difference was 2.63 (CI‐0.97; 6.22), *p* = 0.15 (Table 2 and Table 3). In the per protocol analysis the mean difference between intervention and control groups regarding fatigue at 4 weeks was 4.04 (95% CI 0.14: 7.93), *p* = 0.04 and at 12 months mean difference was 4.29 (95% CI 0.44; 8.14), *p* = 0.03 (Table 3).

**TABLE 2:** Fatigue, quality of life and pain at baseline and follow up at 4 weeks and at 12 months, mental recovery at 4 weeks and 12 months follow‐up.

	Characteristic	Intervention	Control
		*N* = 250	*N* = 268
**Fatigue**	Baseline	65 (50, 80)	70 (50, 80)
median (interquartile range)	missing (*n*)	20	13
	4 weeks postoperatively	60 (40, 75)	55 (35, 75)
	missing (*n*)	31	24
	12 months postoperatively	75 (55, 85)	70 (55, 85)
	missing (*n*)	8	7
**Quality of life**	Baseline	74 (60, 80)	75 (60, 86)
median (interquartile range)	missing (*n*)	30	29
	4 weeks postoperatively	75 (55, 85)	70 (50, 85)
	missing (*n*)	39	22
	12 months postoperatively	80 (70, 90)	80 (70, 90)
	missing, n	9	5
**pain**	Baseline	3 (2, 4)	3 (2, 5)
median (interquartile range)	missing (*n*)	145	152
	4 weeks postoperatively	3 (1, 4)	3 (2, 4)
	missing (*n*)	128	134
	12 months postoperatively	3 (2, 5)	3 (2, 5)
	missing (*n*)	137	157
**Mental recovery**, Number of participants (%)	4 weeks postoperatively		
	Answer alternatives		
	Not relevant, I do not feel recovered	7 (3%)	14 (6%)
	I feel 25% recovered	18 (9%)	19 (8%)
	I feel 50% recovered	34 (16%)	27 (11%)
	I feel 75% recovered	64 (30%)	75 (30%)
	I feel fully recovered	89 (42%)	112 (45%)
	missing (*n*)	38	21
	12 months postoperatively		
	Answer alternatives		
	Not relevant, I do not feel recovered	3 (1%)	4 (2%)
	I feel 25% recovered	6 (3%)	5 (2%)
	I feel 50% recovered	12 (5%)	19 (7%)
	I feel 75% recovered	75 (31%)	82 (31%)
	I feel fully recovered	147 (60%)	151 (58%)
	missing (*n*)	7	7

**TABLE 3:** Difference between intervention and Control groups in Fatigue, quality of life and pain.

	Postoperatively follow ups	Intention‐to‐treat		per protocol	
		Differences and 95% CI	*p*	Differences and 95% CI	*p*
Fatigue	4 weeks	3.75 (0.01, 7.49)	0.05	4.04 (0.14, 7.93)	0.04
	12 months	2.63 (−0.97, 6.22)	0.15	4.29 (0.44, 8.14)	0.03
Quality of life	4 weeks	1.38 (−2.17, 4.92)	0.45	1.25 (−2.49, 4.99)	0.51
	12 months	0.14 (−3.25, 3.54)	0.93	2.09 (−1.57, 5.75)	0.26
pain	4 weeks	−0.18 (−0.83, 0.48)	0.59	−0.35 (−1.06, 0.37)	0.34
	12 months	−0.07 (−0.72, 0.58)	0.84	−0.54 (−1.30, 0.21)	0.16

*Note:* Linear mixed models were used to assessed differences in fatigue, quality of life, and pain at 4 weeks and 12 months. The binary treatment variable (control/intervention) was the primary variable of interest. Models adjusted for baseline outcome, time point (4 weeks/12 months), time‐treatment interaction, tumor site (colon/rectum), neoadjuvant therapy (none/radiotherapy/chemoradiotherapy), and surgery type (open/laparoscopic). Results are presented as mean differences, 95% CIs, and *p*‐values.

Regarding quality of life, no differences were found at 4 weeks (mean difference 1.38 (CI‐2.17; 4.92), *p* = 0.45) or 12 months (mean difference 0.14 (CI‐3.25; 3.54), *p* = 0.93) respectively (Table 3). Pain did not differ at 4 weeks (mean difference −0.18(CI‐0.83; 0.48), *p* = 0.59) or 12 months (mean difference −0.07(CI‐0.72; 0.58), *p* = 0.84) (Table 3). Similarly, the per protocol analyses did not show any differences in quality of life or pain (data not shown).

For mental recovery, a shift towards feeling more recovered was noticed over time in both groups, but no significant differences between groups were found.

## 4 Discussion

In this exploratory analysis of secondary outcomes in a pragmatic randomized study, patients with colorectal cancer randomized to a structured physical activity intervention reported lower fatigue scores at short‐term follow‐up compared with patients receiving usual care. Among patients who achieved per‐protocol compliance with the intervention, a similar pattern was observed at long‐term follow‐up, although this subgroup analysis is subject to selection bias and should be regarded as hypothesis‐generating.

These results represent exploratory analyses of secondary endpoints in the randomized trial PHYSSURG‐C where the primary endpoint was self‐reported physical recovery, which was not significantly different between groups [5, 19]. The wide confidence intervals, the “border‐line” level of the *p*‐value of the primary analysis of this study in combination with the fact that no corrections for multiple testing were made, are reasons for cautious interpretation of the results.

In a pre‐planned subgroup patients who performed the intervention as intended and reported in diaries were analyzed (per protocol). The intervention was individualized and thus this subgroup would not necessarily be composed of patients who were “fitter” at baseline, rather that the result of this analysis could represent a factual outcome of the intervention, when performed as intended. Other outcomes in the per protocol subgroup such as recovery did not differ from the intention to treat cohort either at 4 weeks or 12 months [5, 19]. The hypothesis that medium level physical activity could positively affect outcomes such as recovery or fatigue after colorectal cancer treatment was in part based on earlier findings that a habit of light‐ medium physical activity decreased the risk for postoperative complications after colorectal cancer treatment compared to no physical activity [20]. Testing an intervention of non‐supervised physical activity of medium level aerobic exercise for 2.5‐3 h per week was chosen as a pragmatic model, which could more easily be implemented in routine care if found efficacious, compared to supervised, intensive and multimodal programs. The patients kept a diary of physical activity during the period before surgery, which was used to define the per protocol subgroup. In Sweden a minority of patients diagnosed with colorectal cancer have exercise habits at this level [20]. We have previously reported that fatigue and pain were predictors of global quality of life in a multicenter, international prospective study of patients who underwent surgery for rectal cancer with curative intent [7]. Here we found suggestions, but due to the exploratory nature we cannot definitively confirm, that after an intervention of medium level physical activity the intervention group felt less fatigue compared to controls with a reported mean differences of 3.75 which is within the clinically meaningful difference of RAND‐36 suggested to be 3–5 points for the vitality scale [21]. However, the lower end of the confidence interval was well below the clinically meaningful range, and as such clinical relevance is uncertain. Using the vitality scale in a validated instrument (RAND‐36) should decrease the risk of influencing the outcome by subjectivity in the perception of fatigue. There were no differences regarding quality of life using EuroQol ‐ 5Dimensions, where a clinically meaningful difference has been described as 7–10 points (scale 0–100) [22]. The primary outcome of PHYSSURG‐C was feeling physically recovered, which did not significantly differ between intervention and control (5). We have not explored associations between the outcomes “physical recovery” and “fatigue”.

In a randomized trial testing exercise training in patients treated by adjuvant chemotherapy after breast cancer surgery, a decrease in fatigue larger than in the present study and a stability in pain levels were found [10, 23]. The intervention in that trial was 16 weeks of supervised high intensity training, as opposed to this trial, which was unsupervised, of medium intensity and for a shorter period, 2 weeks pre‐ plus 4 weeks postoperatively. The difference between the two training programs could be factors explaining differences in effect size for fatigue and pain, as could the fact that both men and women were included in our trial. The type of cancer is also a possible factor behind the differences in outcomes, as the surgical trauma is more pronounced after colorectal cancer surgery. The symptom cluster connected with fatigue also differs between breast and colorectal cancer, with pain, appetite loss and nausea being more pronounced factors for patients with colorectal cancer [24]. Different instruments to measure fatigue, pain and quality of life were used, which probably increased difficulties of comparing outcomes.

Prehabilitation is not a strictly defined concept. The term can be used for additional physical activity alone but with varying levels of intensity, unsupervised or supervised [5, 10]. Trials randomizing frail patients have used prehabilitation as a multimodal program including exercise, dietary supplementation and/or psychological support with differences in program and delivery [3, 4] Some have reported improved outcomes [3]. whereas other trials reported no differences between intervention and control groups [4]. Few trials have included a representative cohort of patients with colorectal cancer, but recently a lower risk for at least one serious complication after four weeks of high intensity training was reported [25]. However, there were limitations regarding external validity with inclusion selection and a 22% recruitment rate, ending inclusion before full accrual [25]. As reported earlier we found no effect on complications by the intervention in our trial in the short or long term [5, 19]. In a cohort study, nested in the RCT, we found a lower burden of complications in patients who at baseline (preoperatively) reported more intense exercise habits [20] in line with a prospective cohort study regular physical training before diagnosis was associated with lower risk of postoperative complications [26]. The question of how to improve the average patient's probability to have an uncomplicated and smooth postoperative recovery after surgery for colorectal cancer is still unclear. A recent meta‐analysis concluded that there is not enough evidence for the implementation of general prehabilitation, mainly due to differences in prehabilitation programs (content and duration) and differences in assessment of outcomes [2].

The strengths of PHYSSURG‐C include randomization, multicenter design, and full accrual of a large cohort. One limitation was a relatively large attrition rate in the intervention group. The unsupervised exercise could be regarded as a limitation, as well as the short period of the intervention which was chosen to fit within the time limit from diagnosis to start of treatment (28 days at the time) set by the Swedish authorities in National Cancer Care Pathways. One further limitation of this study was the secondary nature of the outcomes. The sample size for the trial was calculated with the primary outcome in view and thus the analyses of secondary outcomes could be underpowered.

Altogether, an intervention of the magnitude as described did not change feeling recovered four weeks or 12 months after surgery [5, 19], or perception of pain, quality of life, or mental recovery. As emphasized, the results regarding fatigue in this study should be interpreted cautiously due to the nature of the study. Put in a perspective of reported effects of different programs for prehabilitation by physical activity, this could be taken as an indicator that the intensity and/or duration of this intervention was inadequate, and/or that unsupervised training should rather be supervised [2, 3, 4]. In a Swedish context, we have found that 70%–85% of the population diagnosed with breast or colorectal cancer had habits of low to hardly any physical activity; thus, WHO recommendation levels would represent a substantial increase (5,28). The question of prehabilitation before surgery needs further evidence from new, well‐designed, and randomized trials, as evidence for general implementation of any specific program of prehabilitation is still lacking. Obviously, unsupervised interventions without need for extra equipment or frequent visits to health care facilities for patients would be preferable if delivering improved recovery. One way forward, still saving resources for health care and patients, could be supervised training in on‐line group sessions [23].

## Author Contributions

**Kevin Afshari:** conceptualization, methodology, writing – original draft. **Anette Wedin:** investigation, writing – review and editing. **Eva Haglind:** conceptualization, investigation, funding acquisition, methodology, writing – review and editing, project administration, resources. **Eva Angenete:** methodology, writing – review and editing. **Aron Onerup:** conceptualization, methodology, writing – review and editing, investigation, funding acquisition. **Carolina Ehrencrona:** conceptualization, methodology, data curation, writing – review and editing. **Ying Li:** methodology, formal analysis, data curation, writing – review and editing.

## Funding

This work was supported by AFA Insurance 150072, the Swedish state under the agreement between the Swedish govrenment and the couty councils, the ALF agreement ALFGBG‐718221, ALFGBG‐4307771, ALFGBG‐493341, ALFGBG‐784821. Swedish Cancer Society, CAN 2016/519, Pj2019/190303, Pj22 2006. Stiftelsen Assar Gabrielssons Fond, FB19‐07, FB 16‐95. Anna‐Lisa and Bror Björnsson's foundation. Doktor Felix Neuberghs Stiftelse, 2017‐ 347. Göteborgs Läkaresällskap, GLS‐688001, GLS‐778731, GLS‐883991. Lions Cancer Foundation West, 2019:6. Stiftelsen Mary von Sydows, född Wijk, donationsfond, 1216. Svenska Läkaresällskapet, SLS‐499811.

## Disclosure

Permission to Reproduce Data from Other Sources: The Swedish ColoRectal Cancer Register (SCRCR) permitted for the trial to retrieve specified clinical data for use in the trial.

## Ethics Statement

The Regional Ethics Board in Gothenburg approved the trial (2014‐10‐30, DNR:597–14).

## Consent

All patients were informed about the trial (oral and written) before consenting.

## Conflicts of Interest

The authors declare no conflicts of interest.

## Data Availability

The data that support the findings of this study are available on request from the corresponding author. The data are not publicly available due to privacy or ethical restrictions.
